# Extracellular vesicles produced by NFAT3-expressing cells hinder tumor growth and metastatic dissemination

**DOI:** 10.1038/s41598-020-65844-x

**Published:** 2020-06-02

**Authors:** Lívia Cardoso Bueno de Camargo, Frédéric Guaddachi, David Bergerat, Nadia Ourari, Lucie Coillard, Veronique Parietti, Morgane Le Bras, Jacqueline Lehmann-Che, Sébastien Jauliac

**Affiliations:** 1 Université de Paris, Research Saint Louis Institute (IRSL), INSERM HIPI U976, F-75010 Paris, France; 2 Inovarion SAS, (Paris, ile de France, France), Paris, F-75013 France; 3 Université de Paris, Saint-Louis Hospital, Research Saint Louis Institute (IRSL), Département d’Expérimentation Animale (Paris, ile de France, France), Paris, F-75010 France; 4 Molecular Oncology Unit, AP-HP, Hôpital Saint Louis, F-75010 Paris, France

**Keywords:** Cancer, Breast cancer

## Abstract

Metastases are the main cause of cancer-induced deaths worldwide. To block tissue invasion, development of extracellular vesicles (EVs) as therapeutic carriers, appears as an exciting challenge. To this aim, we took advantage of the anti-invasive function of NFAT3 transcription factor we identified previously in breast cancer and addressed the opportunity to transfer this inhibitory function by EVs. We show here that EVs produced by poorly invasive NFAT3-expressing breast cancer cell lines are competent to block *in vitro* invasion of aggressive cancer cells from different origins and, in cooperation with macrophages, inhibit cell proliferation and induce apoptosis. Moreover, this inhibitory effect can be improved by overexpression of NFAT3 in the EVs-producing cells. These results were extended in a mouse breast cancer model, with clear impact of inhibitory EVs on tumor growth and metastases spreading. This work identifies EVs produced by NFAT3-expressing breast cancer cells as an anti-tumoral tool to tackle cancer development and metastases dissemination.

## Introduction

Cancer is one of the leading causes of morbidity and mortality worldwide with an expected increasing number of new cases in the next decades. Regardless of the primary site, the leading cause of death is the formation of metastases. Despite the development of new strategies, efficient treatments are still needed especially for so-called “aggressive” cancers with a high rate of metastases formation (i.e. triple negative breast cancer and pancreatic cancer). Innovative therapeutic approaches, guided by emergent basic science discoveries, are urgently needed to manage or to limit metastatic spreading and distant tissues invasion.

Among the promising therapeutic approaches, the extracellular vesicles (EVs), that usually play roles of intercellular communication mediators, are now thought to be used for drug and anti-tumor therapies delivery^1,2^. If EVs have been proposed as biomarkers of cancer progression and associated with a pro-tumoral role^3,4^, they also represent an attractive alternative to cell therapy, with possible genetic and trophic material transfer to receptor cells, without the risks inherent to the injection of whole living cells. To this end, EVs have already been tested in humans through phase I and II clinical trials, demonstrating safety in variable indications^1^. Rheenen’s group was the first to reveal that EVs have the ability to transmit characteristics of the EV-producer cells *in vivo* to the recipient cells in a breast cancer^5^ and melanoma mice models^6^.

Considering the metastatic players in breast cancer biology, we have previously demonstrated the role of NFAT transcription factors in the dissemination of metastases. We demonstrated that the transcription factor NFAT1 (NFATc2) exerts a pro-invasive function, whereas NFAT3 (NFATc4) has anti-invasive properties limiting the aggressiveness of primary NFAT3-expressing luminal breast cancer cells^7–10^. Since then, several publications have highlighted the critical role of NFAT transcription factors in tumorigenesis in many other cancers (melanoma, pancreas and lung)^11–13^.

Therefore, based on EVs knowledge and on our previous work on NFAT functional roles in metastasis, we aimed to transfer the anti-invasive properties of NFAT3 isotype to tackle cancer development and/or metastatic propension.

Thus, in the present study, we evaluate the use of EVs as endogenous mediators to convey NFAT3 inhibitory properties and target cancer cells both *in vitro* and *in vivo*. Indeed, we show that EVs produced by low invasive luminal breast cancer cells are fully competent to inhibit both cell invasion *in vitro* of cancer cells from different origins and *in vivo* metastases formation in a mice model of breast cancer. Furthermore, besides blocking metastases arising, we demonstrate *in vitro* that these EVs are strong inhibitors of tumor growth in cooperation with macrophages. Strikingly, these EVs inhibitory effects rely on the expression of NFAT3 by EVs-producing cells, yet without any detectable transfer of NFAT3 to the recipient cells. To note, increase of NFAT3 expression in the EVs-producing cells appeared to be sufficient to significantly enhance EVs inhibitory function both *in vitro* and *in vivo*. Together, these results provide fundamental basis to develop and potentiate new EVs-derived therapeutic tools to treat aggressive cancers.

## Results

### EVs produced by poorly aggressive luminal breast cancer cells have anti-invasive properties *in vitro* on different cancer cell types

Having shown that NFAT3, significantly more expressed in luminal breast cancer, inhibits breast cancer cell invasion^9^, we evaluate here the possibility that EVs produced by luminal breast cancer cells might be competent to transfer this inhibitory capacity by NFAT3 to triple negative breast cancer cells lines. To this end EVs were isolated from conditioned medium of different cell lines, purified by the classical ultracentrifugation method and characterized by specific EV markers CD63, CD81 and Calnexin (Fig. S1). The size and concentration of MDA-MB-231 and T-47D EVs were determined by NTA (Nanoparticle Tracking Analysis) allowing to estimate the amount of EVs per producing cells (Fig. S1A).

**Figure 1:**
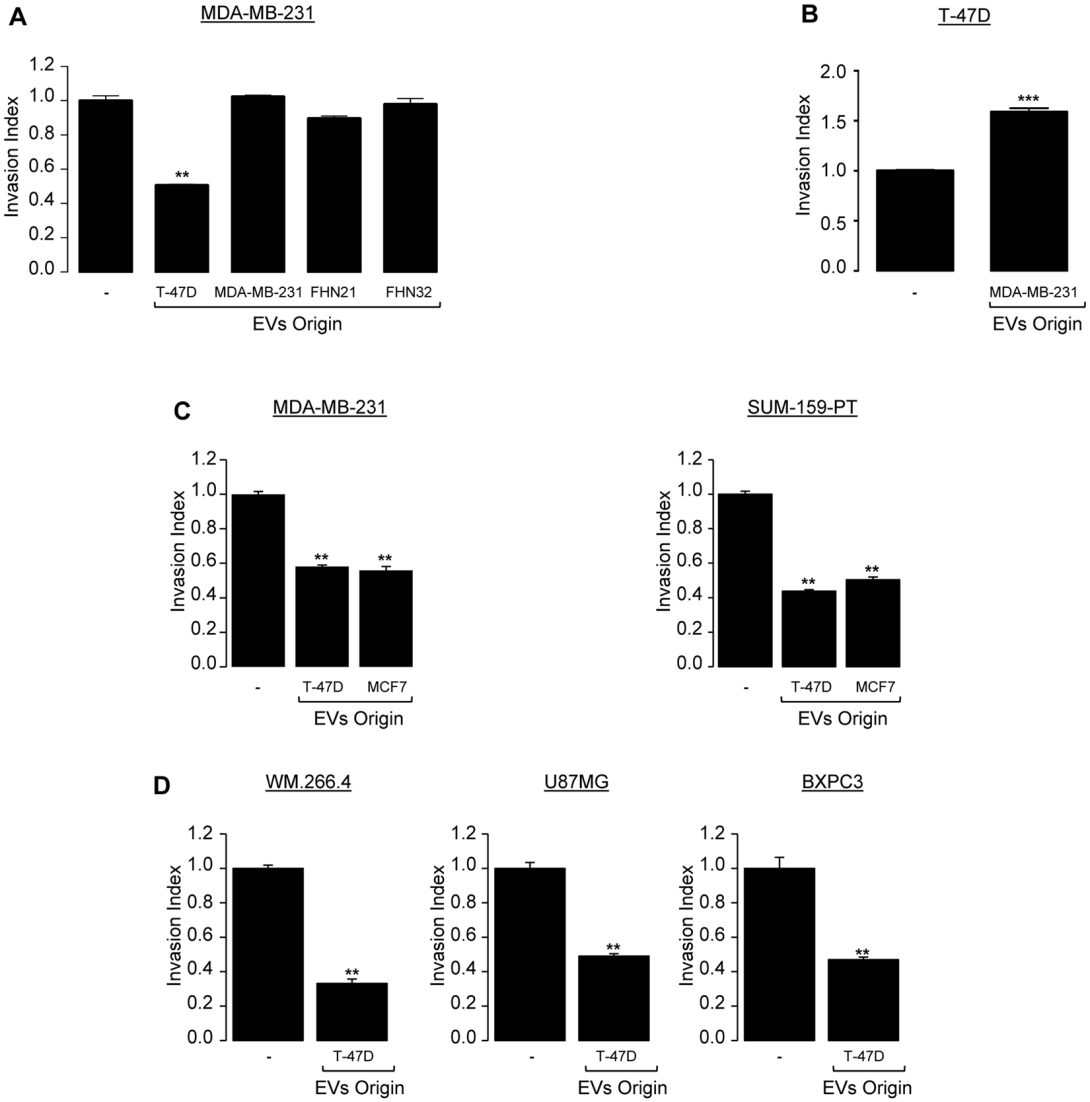
EVs produced by poorly aggressive luminal breast cancer cells exert anti-invasive properties *in vitro* on different types of cancer cells. (**A**) Highly invasive triple negative breast cancer cells MDA-MB-231 were serum starved for 24 h and left untreated or were treated the following day with 3 × 10^8^ pp/mL EVs isolated from by WT T-47D; from WT MDA-MB-231 or from 2 different female primary human dermal fibroblasts (FHN21, FHN32) and subjected to *in vitro* invasion assay for 6 h. Data from one representative experiment of two independent experiments is shown, all data are shown as mean ± SEM (n = 3 technical replicates; **p < 0.005). (**B**) Poorly invasive luminal breast cancer cells T-47D were serum starved for 24 h and left untreated or were treated the following day with 3 × 10^8^ pp/mL EVs produced by WT MDA-MB-231 subjected to *in vitro* invasion assay for 24 h. Data from one representative experiment of two independent experiments is shown, all data are shown as mean ± SEM (n = 3 technical replicates; ***p < 0.001, compared to the untreated cells). (**C**) Highly invasive triple negative breast cancer cells MDA-MB-231 (left panel) and SUM-159-PT (right panel) were serum starved for 24 h and left untreated or treated the following day with 3 × 10^8^ pp/mL EVs produced by WT T-47D or by WT MCF7 and subjected to an *in vitro* invasion assay for 6 h. Data from one representative experiment of two independent experiments is shown, all data are shown as mean ± SEM (n = 3 technical replicates; **p < 0.005, compared to the untreated cells). (**D**) Highly invasive melanoma (WM.266.4), glioblastoma (U87MG) and pancreatic cancer cells (BXPC3) were serum starved for 24 h and left untreated or were treated the following day with 3 × 10^8^ pp/mL EVs produced by WT T-47D and subjected to *in vitro* invasion assay for 6 h. Data from one representative experiment of two independent experiments is shown, all data are shown as mean ± SEM (n = 3 technical replicates; **p < 0.005, compared to the untreated cells). For all data, the invasion index is calculated as a proportion of the number of invasive cells in treated wells compared to the number of invasive cells in the control well (−) arbitrarily set to 1.

To study their potential effect on the invasive capacity of triple negative breast cancer cell lines, we first treated the triple negative MDA-MB-231 breast cancer cells with EVs produced by luminal T-47D breast cancer cells. As controls, we tested on the same cell line the effect of EVs produced by MDA-MB-231 or by normal human fibroblasts originated from two different healthy donors (FHN21, FHN32) (Fig. 1A). Among the different EVs produced, only those originated from T-47D cells were reproducibly efficient in inhibiting MDA-MB-231 cell invasion compared to the EVs from other sources (Fig. 1A). Conversely, EVs produced by highly invasive MDA-MB-231 cells were able to significantly enhance T-47D cell invasion (Fig. 1B) as previously reported *in vivo* by Zomer *et al*.^5^. To confirm the inhibitory effect of the T-47D – produced EVs, we showed that these EVs were reproducibly able to inhibit the invasion of SUMP-159-PT, another triple negative breast cancer line (right panel, Fig. 1C). Moreover, this anti-invasive effect was not specific to the EVs produced by T-47D cells since EVs produced by MCF7, another luminal breast cancer cell line, were shown to be as efficient in blocking cell invasion of both MDA-MB-231 and SUM-159-PT triple negative breast cancer cell lines (left panel, Fig. 1C). These results demonstrate for the first time the specificity and capacity of EVs produced by luminal breast cancer cells to inhibit the invasion of highly aggressive triple negative breast cancer counterparts.

Next, we explored whether this inhibitory effect of T-47D-produced EVs could have a broader spectrum by examining their effect on the cell invasion of other aggressive cancer cell lines from melanoma (WM.266.4), glioblastoma (U87MG) or pancreatic origin (BXPC3). Indeed, results presented in Fig. 1D unveil that the anti-invasive function of EVs produced by T-47D cells was not restricted to highly invasive breast cancer cells but could be extended to other highly aggressive cancer cells from various origins. To note, EVs had no impact on cell proliferation and apoptosis (Fig. S1B). Altogether, these results pinpoint that EVs produced by luminal breast cancer cell lines are fully proficient in impairing highly aggressive cancer cells invasive capacity *in vitro*.

### The anti-invasive capacity of EVs produced by T-47D is linked to the expression of NFAT3 in T-47D producing cells

**Figure 2:**
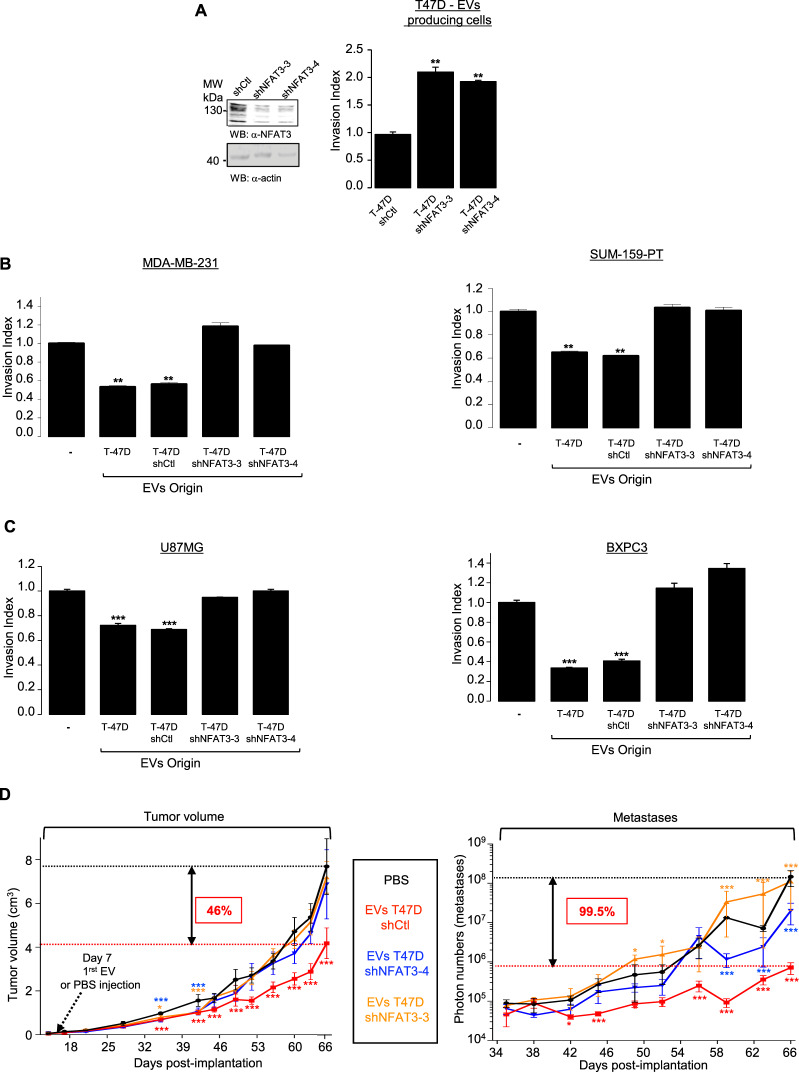
NFAT3 expression in EVs-producing cells is required to confer anti-invasive capacity *in vitro* and anti-tumoral and anti-metastatic functions to EVs *in vivo*. (**A**)  Satble clones expressing either a shRNA control (T-47D shCtrl) or two independents NFAT3 shRNA (T-47D shNFAT3-3, T-47D shNFAT3-4). Left panel: Whole cell lysates were revealed by an anti-NFAT3 (α-NFAT3) and normalization was done by revelation with an anti-actin (α-actin) on the same blot after cutting the membrane. Right panel: T-47D shCtrl, shNFAT3-3 or shNFAT3-4 were serum starved for 24 h and the following day subjected to an *in vitro* invasion assay for 24 h. Data from one representative experiment of three independent experiments is shown, all data are shown as mean ± SEM (n = 3 technical replicates; **p < 0.005, compared to the untreated cells). (**B**) Highly invasive MDA-MB-231 or SUM-159-PT cells were serum starved and pre-treated or not with 3 × 10^8^ pp/mL EVs produced by WT T-47D (T-47D) or T-47D shCtrl, T-47D shNFAT3-3, T-47D shNFAT3-4 and tested for their invasion for 6 h. Data from one representative experiment of three independent experiments is shown, all data are shown as mean ± SEM (n = 3 technical replicates; **p < 0.005, compared to the untreated cells). (**C**) Glioblastoma U87MG cells or pancreatic cancer cells BXPC3 were treated as described above and tested for their invasion for 6 h. Data from one representative experiment of three independent experiments is shown, all data are shown as mean ± SEM (n = 3 technical replicates; **p < 0.005, ***p < 0.001, compared to the untreated cells). For all data, the invasion index is calculated as a proportion of the number of invasive cells in treated conditions compared to the number of invasive cells in the control condition (−) arbitrarily set to 1. (**D**) 5 × 10^5^ MDA-MB-231 cells (D3H2LN-LUC) were injected into the left Fat Pad of each 6-weeks-old female mouse Athymic Nude mice. Tumor volume: Tumor growth is presented as the mean tumor volume (cm^3^) ±SEM, from mice injected weekly intra tumor with PBS or with EVs T-47D shCtl (5 × 10^9^ pp) or EVs T-47D shNFAT3-3, EVs T-47D shNFAT3-4, 7 days after cell xenotransplantation, 8 mice per groups. Metastases: Beginning day 35, weekly, bioluminescent images were acquired on the IVIS system to quantify the mean of photons flux produced by the metastatic cells. Metastases quantification is presented as the mean photon flux produced by the metastatic cells. Data from one representative experiment of two independent experiments is shown, all data are shown as mean ± SEM (n = 8 technical replicates;, *p < 0.05 **p < 0.005 and ***p < 0.001, compared to the PBS treated mice group).

To decipher the mechanisms responsible for the anti-invasive effect of the EVs generated by the luminal breast cancer cells, we took advantage of our previous work showing that luminal breast cancer cells specifically express NFAT3, an anti-invasive isotype of the NFAT transcription factors family^9^. We hypothesized that the endogenous NFAT3 expressed in T-47D cells might confer its inhibitory function to the derived-EVs. To validate this hypothesis, we generated stable T-47D cell lines expressing lentiviral constructs encoding either a shRNA control (T-47D shCtl) or two different shRNAs targeting endogenous NFAT3 (T-47D shNFAT3-3, T-47D shNFAT3-4) that reduced up to 60% of NFAT3 expression compared to the shCtl expressing cells (Fig. 2A, left). As previously shown^9^, we confirmed that reducing endogenous NFAT3 expression in T-47D cells increased by two times their invasive capacity (Fig. 2A, right). We produced EVs from these different T-47D clones and verified their homogeneity in size by NTA (Fig. S3). We did not observe any effect of NFAT3 downregulation on EVs production efficiency (Fig. S3A). In addition, and conversely to what we observed for T-47D-produced EVs, T-47D shNFAT3-3 and T-47D shNFAT3-4 produced EVs were ineffective to inhibit cell invasion neither of triple negative breast cancer cell lines (Fig. 2B), nor glioblastoma or pancreatic cell lines (Fig. 2C). These results demonstrate that EVs produced by poorly invasive luminal breast cancer cells are fully competent at inhibiting aggressive cancer cells invasion and rely on NFAT3 expression in the EVs producing cells.

### NFAT3-expressing luminal breast cancer cells produced EVs are fully competent at inhibiting *in vivo* breast tumor growth and metastases burden

Since cell invasion is critical for metastases spreading *in vivo*, we sought to examine whether these anti-invasive EVs might also be competent in preventing metastases burden in a murine breast cancer model. To this end we xenografted MDA-MB-231-luciferase positive cells (D3H2LN-LUC) in mice fat pad. We generated 4 groups of mice that were subjected to a weekly intra-tumor injection, starting 1 week after cells implantation, with either PBS as a control or the different EVs originated from T-47D shCtl cells or T-47D shNFAT3-3/shNFAT-4 cells. Tumor growth was evaluated once a week for each group by caliper measurements and after one month, metastases apparition was quantified once a week by luminescence with an IVIS *in vivo* imaging system.

During the first 30 days tumor growth was equivalent in all the 4 mice groups (Fig. 2D, Tumor volume, day 0–28). Strikingly, from day 42, only the group treated with the EVs produced by T-47D shCtl cells showed a significant reduction of tumor growth, that pursue as far as the end of the experiment. At final point, we observed a reduction of 46% of average tumor size compared that of mice groups injected either with PBS or with EVs originated from T-47D shNFAT3 cells (Fig. 2D, Tumor volume, day 42–66). Although interesting, these *in vivo* results do not correlate with the absence of EVs effect on proliferation and apoptosis seen *in vitro* (Fig. S1B ). This suggest that other mechanisms are engaged upon EVs treatment to favor the inhibition of tumor growth in physiological mode.

Moreover, we completed this experimental set by analyzing metastasis burden, one month after cancer cells injection. As shown in Fig. 2D (right panel, Metastases), the group of mice injected with the EVs produced by T-47D shCtl cells present a significative inhibition of metastases burden, from day 42 until the end of the experiment, to reach 99,5% of inhibition compared to the group of mice injected with either PBS or EVs produced by T-47D shNFAT3-3/shNFAT3-4. Representative images of metastases burden are shown for day 59 in Fig. S3B.

As a complement of our *in vitro* results, these later data show that EVs produced by NFAT3-expressing low invasive breast cancer cells can inhibit metastases spreading but also tumor growth of an aggressive triple negative breast cancer cell line.

### EVs from NFAT3-expressing cells require macrophages to inhibit cell growth and to induce apoptosis in breast cancer cells

**Figure 3:**
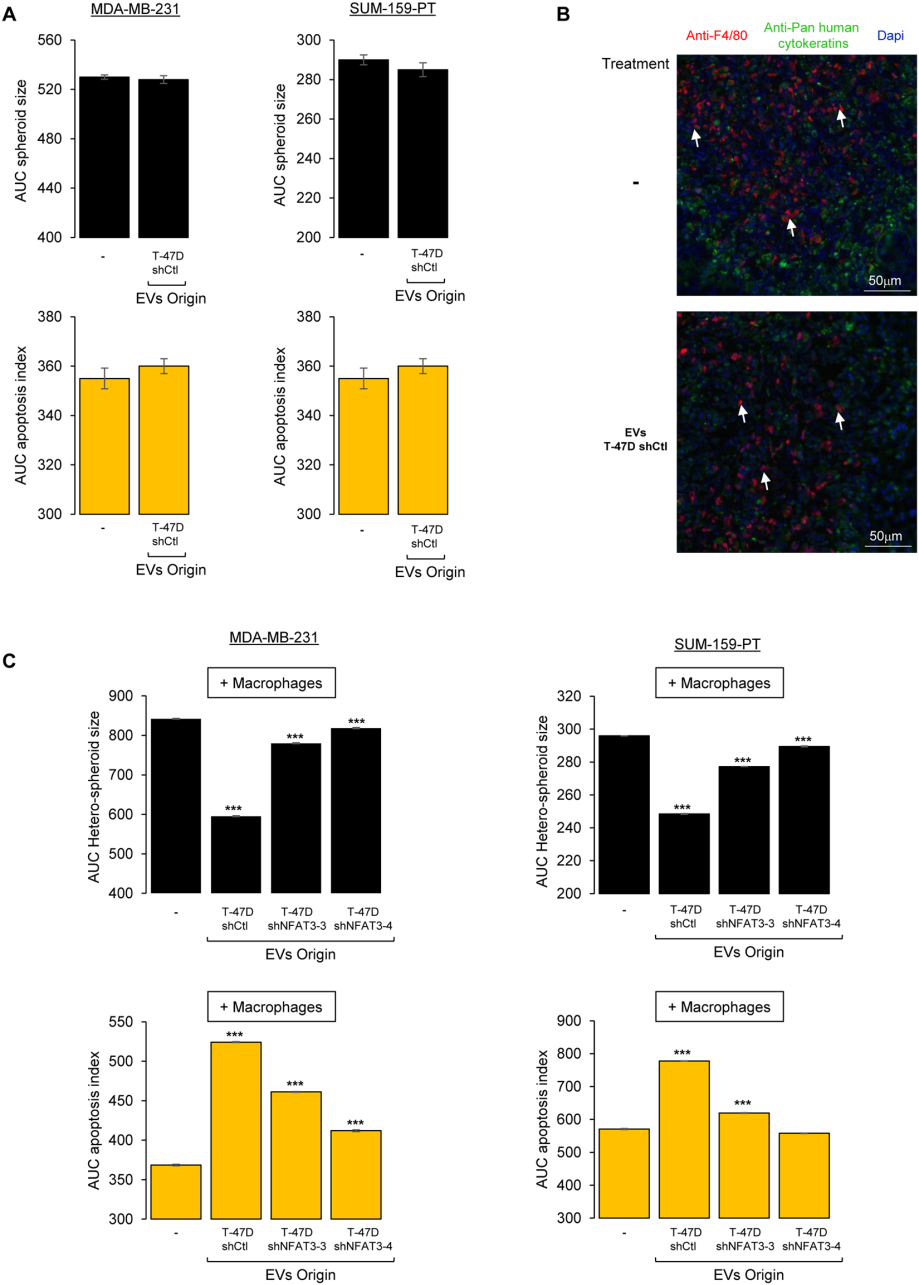
EVs from NFAT3-expressing cells inhibit cell growth and increase apoptosis in cancer cells in cooperation with macrophages. (**A**) MDA-MB-231 cells (left panel) and SUM-159-PT cells (right panel) were plated in medium with 3% Matrigel in 96-well ultra low attachment plates for three days to allow the formation of the spheroids formed. Medium was then added to each condition containing the apoptosis indicator (fluorescent caspase 3/7 substrate) supplemented with medium and containing or not the different EVs (3 × 10^8^ pp/mL) as indicated. Size of the spheroids and appearance of green fluorescence (apoptosis) was recorded every 2 h on an INCUCYTE device for 4 days. Data are represented as the AUC of the spheroid size (upper panels) and apoptosis (lower panels) over 96 h. Data from one representative experiment of three independent experiments is shown, all data are shown as mean ± SEM (n = 3 technical replicates). **(B)** Frozen tumor tissues sections of mice xenografted with MDA-MB-231 cells (D3H2LN-LUC) and treated with PBS (−) or EVs T-47D shCtl were co-labelled with Dapi and specific antibodies to mouse macrophages (anti-F4/80 antibody) and to detect the tumor cells with an anti-Pan human cytokeratin (arrows indicate infiltrating mouse macrophages). **(C)** Hetero-spheroids containing 33% of the murine macrophage cell line RAW 264.7 with either MDA-MB-231 or SUM-159PT were plated in medium with 3% Matrigel in 96 wells ultra low attachment plates for three days to allow the formation of the hetero-spheroids formed. Then medium was added to each condition containing the apoptosis indicator (fluorescent caspase 3/7 substrate) supplemented with medium containing or not the different EVs (3 × 10^8^ pp/mL) as indicated in the figure. The size of the spheroids and appearance of green fluorescence (apoptosis) were recorded every 2 h on an INCUCYTE apparatus for 4 days. Data are represented as the AUC of the spheroid size (upper panels) and apoptosis (lower panels) over 96 h. Data from one representative experiment of three independent experiments is shown, all data are shown as mean ± SEM (n = 3 technical replicates; ***p < 0.001, compared to the untreated cells).

Even though we were unable to detect anti-proliferative or pro-apotoptic effects of the T-47D-produced EVs *in vitro*, we reproducibly observed an inhibitory effect of these EVs on tumor growth *in vivo*. As for the anti-invasive effects observed, these inhibitory effects on the tumor growth were linked to NFAT3 expression in the EVs-producing cells. Therefore, we questioned the 2D cell culture system previously used and set up a protocol for breast cancer cell spheroids culture (see Mat & Meth section). Cells were seeded and cultured for 3 days in low attachment plates embedded in 3% Matrigel and spheroids size was monitored using the INCUCYTE device. Medium containing the apoptosis indicator (fluorescent caspase 3/7 substrate) was added to cells and supplemented with or without the T-47D-produced EVs. Again, we could not identify any anti-proliferative or pro-apoptotic effects of the EVs in these conditions (Fig. 3A).

To further explain the difference between our *in vitro* and *in vivo* results, we chose to monitor cell microenvironment. Indeed, it has been shown *in vivo*, that the tumoral microenvironment constituted by many cells, such as cancer associated fibroblasts and macrophages, can have a direct impact on tumor growth^14^. In our experimental conditions, this cell contingent might play crucial role and be necessary for the EVs-induced-inhibition of the tumor growth.

Therefore, we evaluated by immunofluorescence the presence of macrophages in the tumor we generated by xenotransplantation of the MDA-MB-231 cells in the fat pad of the athymic nude mice. Human tumor cells were detected based on human cytokeratins expression and mouse macrophages owing to the detection of the F4/80 expression marker. As shown in Fig. 3B, mouse macrophages (anti-F4/80) were found to infiltrate the engrafted tumor (anti-Pan human cytokeratins). No increase of macrophages recruitment within the tumor was observed for mice treated with the inhibitory EVs compared to the untreated mice.

Thus, in order to mimic the tumoral context and to identify the putative role of these infiltrating macrophages in the tumor growth inhibition, we designed co-culture conditions and set up protocol to generate hetero-spheroids containing 2/3 of breast cancer cells (MDA-MB-231 or SUM-159-PT) and 1/3 of the murine macrophage cell line RAW 264.7 embedded in 3% Matrigel. Three days later, medium containing the apoptosis indicator (fluorescent caspase 3/7 substrate), supplemented either with or without the indicated EVs was added. In this cellular system, EVs had no effect on macrophages cell growth while apoptosis was undetectable in this cell line (Fig. S2). While inefficient in our previous settings, (Fig. 3A), in this 3D system complemented with mouse macrophages, EVs T-47D shCtl were fully competent to reduce hetero-spheroids size and to increase apoptosis compared to the untreated cells (Fig. 3C; - and EVs T-47D shCtl). Moreover, the impact on hetero-spheroids growth and apoptosis induction was reversed upon NFAT3 downregulation in T-47D producing cells (Fig. 3C; EVs T-47D shNFAT3/4). These results obtained for both triple negative breast cancer cell lines tested correlate with the *in vivo* effect of EVs on tumor growth and underline the cooperation of EVs with tumoral macrophages to hinder breast cancer cell growth.

### Overexpressing NFAT3 in T-47D EVs-producing cells enhances the anti-invasive function *in vitro*

**Figure 4:**
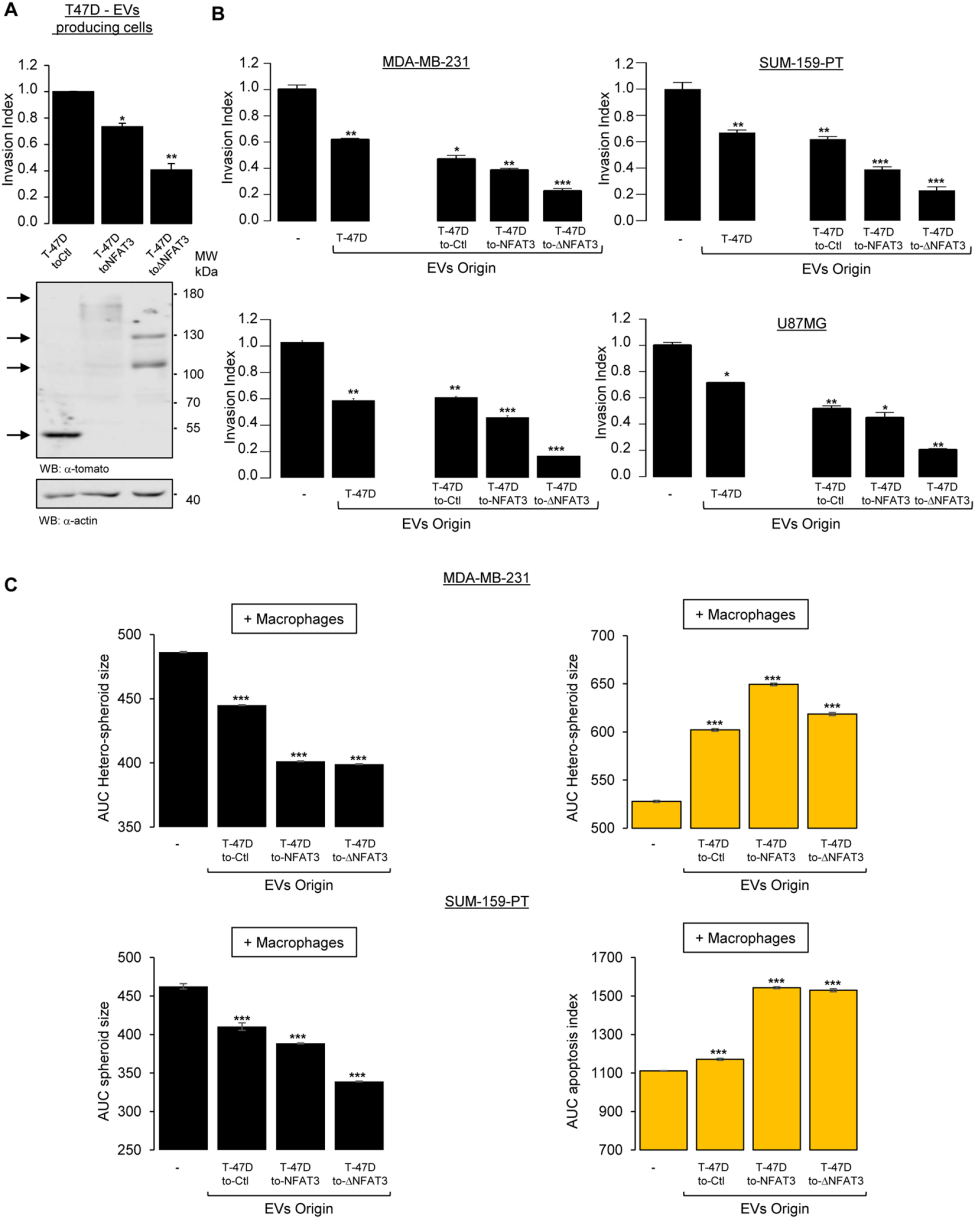
The anti-invasive function of EVs *in vitro* can be further enhanced by overexpressing NFAT3 in T-47D EV-producing cells. (**A**) Stable clones of the T-47D cell line were generated expressing lentiviral constructs encoding either the control vector with a Tomato tag (T-47D toCtl) or the wild type NFAT3 (T-47D toNFAT3) or the active N-terminal deletion mutant of NFAT3 fused to the Tomato tag (T-47D toΔNFAT3). Lower panel: Western blot of T-47D cells stable clones. Whole cell lysates were revealed by an anti-tomato (α-tomato) and normalized by revelation with an anti-actin (α-actin) after stripping on the same blot. Upper panel: T-47D toCtl, T-47D toNFAT3 and T-47D toΔNFAT3 stable clones were serum starved for 24 h and the following day subjected to an *in vitro* invasion assay for 24 h. Data from one representative experiment of two independent experiments is shown, all data are shown as mean ± SEM (n = 3 technical replicates; *p < 0.05, **p < 0.005, compared to the untreated cells). **(B)** Highly invasive MDA-MB-231, SUM-159-PT, BXPC3 and U87MG cells were serum starved and pre-treated or not with 3 × 10^8^ pp/mL EVs produced by WT T-47D or by T-47D toNFAT3, T-47D toΔNFAT3 and T-47D toCtl and tested for their invasive capacity for 6 h. Data from one representative experiment of two independent experiments is shown, all data are shown as mean ± SEM (n = 3 technical replicates; **p < 0.005, p < 0.001, compared to the untreated cells). For all data, the invasion index is calculated as a proportion of the number of invasive cells in treated wells compared to the number of invasive cells in the control well (−) arbitrarily set to 1. **(C)** Hetero-spheroids containing 33% of the murine macrophage cell line RAW 264.7 with either MDA-MB-231 or SUM-159PT were plated in medium with 3% Matrigel in 96 wells ultra low attachment plates for 3 days to allow the formation of the hetero-spheroids. Medium was then added to each condition containing the apoptosis indicator (fluorescent caspase 3/7 substrate) with medium containing or not the different EVs (3 × 10^8^ pp/mL). The size of the spheroids and green fluorescence (apoptosis) was recorded every 2 h on an INCUCYTE apparatus for 4 days. Data are represented as the AUC of the spheroid size (upper panels) and apoptosis (lower panels) over 96 h. Data from one representative experiment of three independent experiments is shown, all data are shown as mean ± SEM (n = 3 technical replicates; ***p < 0.001, compared to the untreated cells).

As EVs therapeutic use is explored for anti-tumor therapies^1,2^, increment of their intrinsic anti-tumoral effects described here would be interesting. As illustrated by the present work, NFAT3 could be a pertinent candidate since inhibition of cell invasion *in vitro* absolutely required NFAT3 expression in EVs producing luminal breast cancer cells (Fig. 2). Then, we asked whether overexpression of wild-type NFAT3 or a constitutively active form of NFAT3 previously described^9^ in these cells would be a way to potentiate the inhibitory capacity of derived EVs. To this end, we generated by lentiviral transduction stable T-47D cell lines expressing either the control vector with a Tomato tag (T-47D toCtl), the wild-type NFAT3 (T-47D toNFAT3) or the active N-terminal deletion mutant of NFAT3 fused to the Tomato tag (toΔNFAT3). After validation by Western Blot (Fig. 4A, lower panel), we confirmed that, compared to empty vector, wild-type NFAT3 transduction (toNFAT3) inhibits T-47D cell invasion, with a stronger effect upon transduction of the active N-terminal deletion mutant of NFAT3 (toΔNFAT3) (Fig. 4A, upper panel). These results are in coherence with previous data obtained after transient transfections^9^. We then isolated EVs from T-47D toCtl, toNFAT3 or toΔNFAT3, validate their size and concentration by NTA (Nanoparticle Tracking Analysis) along with the amount of EVs produced per cell (Fig. S4). We did not observe any impact of NFAT3 overexpression on EVs production efficiency (Fig. S4). To test their effects on the invasive capacity, cell growth and apoptosis of the different aggressive breast cancer cell lines used above. Compared to the effect of EVs T-47D toCtl cells (T-47D toCtl), EVs produced by T-47D toNFAT3 slightly but significantly increase their anti-invasive effect, with a much stronger effect with the EVs produced by T-47D toΔNFAT3 in MDA-MB-231 and SUM-159-PT breast cancer cell lines (Fig. 4B, upper panels). Interestingly, these results were reproducible in BXPC3 pancreatic cancer and U87MG glioblastoma cell lines (Fig. 4B, lower panels). These results show the central role of NFAT3 in the EVs-producing cells.

Therefore, we wondered if these results were specific for T-47D-producing cells and decided to test NFAT3 constructs overexpression in the non-tumoral cell line HEK293T. First, we generated EVs from wild-type HEK293T and tested their effect on MDA-MB-231 cell invasion in comparison with the EVs produced in wilt-type T-47D. Strikingly results presented in Fig. S6A showed that EVs produced in HEK293T cells were as efficient to inhibit MDA-MB-231 cell invasion as the EVs generated by the T-47D cells. Therefore, as shown if Fig. S6B, we evaluated the potential expression of endogenous NFAT3 in HEK293T cells and validated that indeed NFAT3 was specifically expressed in these cells as shown by a NFAT3 siRNA. We then generated stable clones of HEK293T overexpressing the different NFAT3 constructs described above (Fig. S6C). The size and concentration of HEK293T EVs were determined by NTA along with the amount of EVs produced per cell (Fig. S5). We did not observe any effect of NFAT3 overexpression on EVs production efficiency (Fig. S5).

As for the T-47D cell line, overexpression of the NFAT3 constructs in HEK293T cells generated EVs with an enhance anti-invasive capacity underlining once again the central role of NFAT3 expression in EVs-producing cells independently of the cell type (Fig. 6D). Thus, overexpression of the NFAT3 constructs could be a general feature for inducing production of EVs with an enhanced anti-invasive capacity. Confirmed in two different human cell lines, these data emphasize the role of NFAT3 expression in EVs-producing cells for efficient cell invasion inhibition.

We then tested the effect of T-47D toNFAT3/toΔNFAT3 on the *in vitro* 3D cancer cells/macrophages co-culture system described above. EVs generated from both T-47D toNFAT3/toΔNFAT3 cells exert a strongest effect on hetero-spheroids growth inhibition as on apoptosis induction compared to the EVs originated from toCtl cells (Fig. 4C). To note, no transfer of overexpressed NFAT3 protein nor RNA could be detected in recipient cells (data not shown). Altogether, these results confirm the central role of NFAT3 expression in EVs-producing cells for consecutive signalization and global anti-proliferative and anti-metastatic effects and highlight the feasibility of increasing these inhibitory capacities by overexpressing NFAT3 in the EVs-producing cells.

### NFAT3 overexpression in EVs-T-47D producing cells increases anti-tumor properties and inhibit tumor growth on a pre-established tumor model *in vivo*

**Figure 5:**
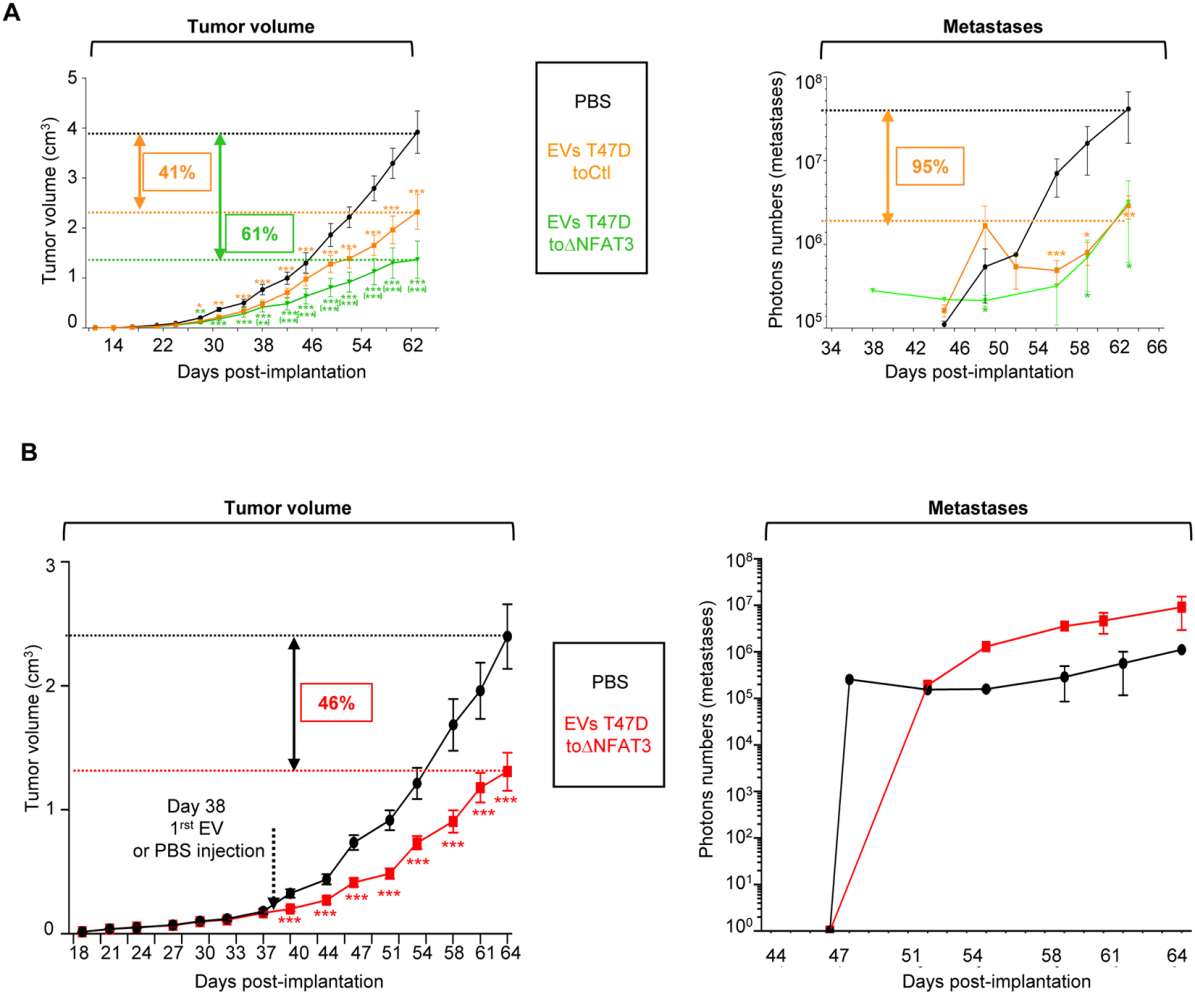
EVs from NFAT3-expressing cells can further increase their anti-tumor effects and are effective in inhibiting tumor growth on a pre-established cancer. (**A**) 5.10^5^ MDA-MB-231 cells (D3H2LN-LUC) were injected into the left Fat Pad of each 6-weeks-old female Athymic Nude mouse. Tumor volume: Tumor growth is presented as the mean tumor volume (cm3) ±SEM, from mice injected weekly intra tumor with PBS or with EVs T-47D toCtl (5 × 10^9^ pp) or EVs T-47D ΔNFAT3, 7 days after cell xenotransplantation, 10 mice per group. Metastases: Beginning day 35, weekly, bioluminescent images (where the primary tumor was shield with a black tissue) were acquired on the IVIS system to quantify the mean photon flux produced by the metastatic cells. Metastases quantification is presented as the mean photon flux produced by the metastatic cells. Data from one representative experiment of two independent experiments is shown, all data are shown as mean ± SEM (n = 10 technical replicates;, *p < 0.05 **p < 0.005 and ***p < 0.001 compared to PBS-injected mice; (**), (***) compared to EVs-T-47D toCtl-injected mice). (**B**). 10^5^ MDA-MB-231 cells (D3H2LN-LUC) were injected into the left Fat Pad of each 6-weeks-old female Athymic Nude mouse. Tumor volume: Tumor growth is presented as the mean tumor volume (cm3) ±SEM, from mice injected weekly intra-tumor with PBS or with 5 × 10^9^ pp EVs T-47D ΔNFAT3, 38 days after cell xenotransplantation, 10 mice in each group. Metastases: Beginning at day 45, weekly, bioluminescent images (where the primary tumor was shield with a black tissue) were acquired on the IVIS system to quantify the mean photon flux produced by the metastatic cells. Metastases quantification is presented as the mean photon flux produced by the metastatic cells. Data from one representative experiment of three independent experiments is shown, all data are shown as mean ± SEM (n = 10 technical replicates; ***p < 0.001, compared to the PBS treated mice group).

As ectopic expression of NFAT3 in EVs-producing cells was suitable to enhance EVs anti-invasive, anti-proliferative and pro-apoptotic effects *in vitro*, we evaluated *in vivo* the anti-tumoral effects of modified EVs. To this end, we established 3 groups of mice that were subjected to weekly intra-tumor injection, starting 1 week after cells implantation, with either PBS as a control or the different EVs originated from T-47D toCtl cells or T-47D toΔNFAT3 cells. Tumor growth was evaluated twice a week for each group by caliper measurements and, after one month, metastases apparition was quantified twice a week by luminescence with an IVIS *in vivo* imaging system. Beginning on day 24, mice injected with the EVs produced by T-47D toΔNFAT3 or by T-47D toCtl showed a similar significant reduction of tumor growth rate, compared to the mice group injected with PBS, lasting for 10 days (Fig. 5A, Tumor volume, day 24–34).

Remarkably, from day 38 to the end of the experiment, if injection of T-47D toCtl-produced EVs exerted by itself an anti-tumor growth effect (41% tumor volume reduction) compared to PBS-treated group, the curtail of the tumor growth was significantly stronger for the group of mice injected with the EVs toΔNFAT3 cells (61% tumor volume reduction) (Fig. 5A, Tumor volume, day 34–62). Nonetheless, despite of the clear inhibition of tumor growth mediated by T-47D toΔNFAT3 produced EVs, these vesicles were not further efficient in preventing metastases apparition, compared to the group of mice injected with the EVs T-47D toCtl (Fig. 5A, Metastases). Indeed, tested EVs T-47D toΔNFAT3 and EVs T-47D toCtl induced a similar reduction (95%) of detected metastases, compared to mice injected with PBS (Fig. 5A, Metastases), illustrating the absence of correlation between the tumor size and the consecutive extend of metastatic dissemination.

To get closer to clinical situations, we further evaluated EVs potential effectiveness on a pre-established tumor *in vivo*. To this end, D3H2LN-LUC cells were xenografted in the fat pad of 2 mice groups, with a measurement of the tumor growth twice a week during 1 month without any EVs injection. After this first month, once the tumor had grown, one group of mice was intra-tumorally injected, once a week, with PBS and the second group with EVs originated from T-47D toΔNFAT3 cells. Tumor growth was evaluated 2 times/week by caliper measurements and metastases apparition was quantified 2 times/week by luminescence with an IVIS *in vivo* imaging system. Strikingly, the results presented in Fig. 5B show that as soon as 2 days after the first EVs injection, the tumor growth was significantly reduced compared to the PBS-treated control group. This slow-down pursued until the experiment ends, to reach a 46% of inhibition (Fig. 5B, Tumor volume), validating the beneficial effect of T-47D toΔNFAT3-EVs to prevent tumor growth. On the opposite, no statistical beneficial effect of delayed EVs injection was observed on metastases apparition (Fig. 5B, Metastases). Globally, these results clearly demonstrate that the inhibitory effect of EVs on tumor growth can be technically improved by overexpressing ΔNFAT3 in T-47D producing cells and was efficient on a pre-established tumor, raising interesting concern in the cancer fight field.

## Discussion

Identifying effective treatments of highly metastatic cancer (i.e. triple negative breast cancer (TNBC) and pancreatic cancer) is the urgent challenge of the next decade. Indeed, TNBC remain the breast cancer subgroup with the least benefit from targeted therapies^15^. Therefore, the early-stage TNBC patients are mainly treated with combinations of taxane and anthracycline chemotherapy with severe side effects and unfortunately a frequent recurrence of the metastases in the first 3 years after surgery^16,17^. These therapeutic situations underline the urgent necessity of finding innovative therapeutic approaches to treat these aggressive cancers. In this context, EVs-based therapy shows an exponential interest with *in vitro* and *in vivo* studies showing promising results^18^.

In our present study, we have shown for the first time that EVs originated from NFAT3-expressing poorly aggressive luminal breast cancer cells are competent to alone inhibit triple breast cancer cell lines invasion and, in cooperation with macrophages, spheroids growth *in vitro*. We verified that these EVs inhibitory capacities were not restricted to the EVs produced by the T-47D cell line as another luminal breast cancer cell line (MCF-7) was able to generate inhibitory EVs too. Moreover, we also established that the inhibitory functions of these EVs were not restricted to the MDA-MB-231 breast cancer cell as another triple negative breast cancer cell line (SUM-159PT) melanoma, pancreatic and glioblastoma cancer cell lines were also similarly affected by incubation with these EVs. In addition, our study extends these results *in vivo* by demonstrating that these early injection of EVs are efficient to inhibit metastases arising and tumor growth in an *in vivo* breast cancer mice model.

The tumor microenvironment is determinant in tumoral progression and among the numerous types of cells presents in this surrounding tissue, macrophages play a central role in tumoral evolution by interacting either directly or indirectly with the tumor cells^14^. We show here that infiltrated macrophages were present within the tumor in our breast cancer mice model and demonstrate, *in vitro*, that the presence of macrophages was compulsory for the EVs to inhibit breast cancer tumor cells growth associated with an increase of apoptosis. These data could be explained by two not exclusive hypotheses, either (i) by an EVs-based sensitization of breast cancer cells to intra-tumoral macrophages killing, or (ii) by a functional interaction between EVs and macrophages to enhance their intrinsic killing capacity. To note compared to data previously reported^19^,We did not reveal in our work any specific M1 or M2 polarization of the macrophages as it has been previously reported^19^.

Mechanistically, we demonstrate the requirement of NFAT3 expression in the EVs-producing cells to generate efficient anti-tumoral EVs. These results could suggest that either NFAT3 protein or NFAT3 mRNA or NFAT3-regulated targets (proteins, RNA, miRNA, for example) need to be transferred by the EVs to the recipient cells to induce anti-tumoral effects. Nonetheless, we were so far unable to detect either the NFAT3 protein nor the NFAT3 mRNA in target cancer cells or EVs, arguing more in favor of NFAT3-regulated targets that might be involved in the anti-invasive/anti-metastatic and anti-proliferative effects of EVs produced by luminal breast cancer cells. These NFAT3-regulated targets have now to be identified and characterized.

Furthermore, we show that these EVs have the capacity to inhibit *in vivo* a pre-established tumor closest to the clinical situation of patients with measurable lesions at the diagnostic. However, in this context, no detectable effects of the EVs on metastases arising were detected. This last observation raises the question of the timing of metastases formation and suggest that the metastatic dissemination might occur early in the tumor’s development, as seen in the patients, before EVs injection as it has been reported by other studies^20,21^. Moreover, these data suggest that rather than eliminating pre-exiting metastases EVs treatment might prevent tumor cells from leaving the primary tumor to colonize other organs.

The anti-tumoral EVs we describe in this study may represent a potential new therapeutic tool to impede breast cancer progression. Indeed, these EVs show an inhibitory effect not only on breast cancer cells but also on other cancer cells, from melanoma, pancreas and glioblastoma origin, emphasizing their pervasive interest for large therapeutic applications. Indeed, we have shown that overexpressing an active form of NFAT3 in EVs-producing cells was a meaningful approach to boost their inhibitory capacities. Moreover, the possibility of using a non-tumorigenic human cell line as the HEK293T to generate enhanced inhibitory EVs might open avenues for potential future developments of production of therapeutic EVs according to GMP (Good Manufacturing Practice). Further studies on the ΔNFAT3-EVs mechanisms of action and ways to enhance their potency are still needed, in particular to understand the molecular determinants present within the EVs and the mechanistic consequences within the recipients’ cancer cells and the microenvironment cells. Associated with the EV-based therapy studies already published, our results emphasize the potential possibility of using EVs with enhanced anti-tumor capacities in cooperation with the immune system, to counteract tumor cell proliferation and subsequent distant invasion of aggressive cancers.

## Methods

### Cell lines culture

MDA-MB-231, T-47D, MCF7, NIH3T3, HEK293T, BXPC3, U87MG, RAW 264.7 cell lines come from the American Type Culture collection. The SUM-159-PT cell line was provided by Alex Toker (Harvard Medical School). MDA-MB-231 D3H2LN-LUC no. CVCLD257) were bought from Perkin Elmer. WM.266.4 was provided by Nicolas Dumaz (Inserm, HIPI U976). MDA-MB-231 and SUM-159-PT cells were cultured in low glucose (1 g/L) DMEM medium. T-47D, MCF7 and BXPC3 cells were cultured in RPMI 1640 medium. shRNA or Tomato transduced T-47D cells were culture in RPMI 1640 medium supplemented with 1.5μg/mL of puromycin (Thermo Fisher Scientific). MDA-MB-231 D3H2LN-LUC were cultured in Eagle’s MEM supplemented with 75ug/mL Zeocin (Invivogen). NIH-3T3, WM.266.4, RAW264.7, HEK293T and U-87MG cells were cultured in high glucose (4,5 g/L) DMEM. All media were complemented with 100 U/mL penicillin and 100μg/mL streptomycin (Thermo Fisher Scientific), an anti-mycoplasma (normocin 100μg/mL, Invivogen) and 10% fetal bovine serum (FBS) (PAN BIOTECH); 10% newborn calf serum (PAN BIOTECH) was added to the medium of NIH3T3. All cell lines were routinely tested for the absence of mycoplasma contamination by PCR. The anti-mycoplasma drug (normocin) and the puromycin were omitted in all EVs productions and experiments.

### Generation and characterization of stable cell lines

To produce T-47D shCtl, T-47D shNFAT3-3 and T-47D shNFAT3-4 stable cell lines, T-47D were stably infected with shRNAs cloned in the pGIPZ lentiviral vector obtained from Dharmacon (sequences: shNFAT3-3: CAATGAACACCACCTTGGA; shNFAT3-4: AGTCTCAGGGAACATCCGC). Lentiviral shRNAs were produced in HEK293T cells using the trans-lentiviral shRNA packaging kit with calcium phosphate (Dharmacon) following the manufacturer’s instructions. Viral particles were collected and concentrated. T-47D cells were infected, selected in presence of 1.5μg/mL of puromycin, and sorted by flow cytometry on the basis of GFP expression. Effective down-regulation of the endogenous NFAT3 was validated by Western blot (anti-NFAT3: 1:1000, no. AB 2267268, Thermo Fisher Scientific; anti-NFAT3: 1:1000, no. AB 650208, Santa-Cruz Biotechnology).

To produce T-47D toCtl, T-47D toNFAT3 and T-47D toΔNFAT3 stable cell lines, T-47D were stably infected either by vontrol viral particles or expressing NFAT3 or ΔNFAT3 particles (from cDNA cloned in the pLVX lentiviral vector tagged with a td-Tomato epitote obtained from Clontech). All the cloned sequences were verified by sequencing. Lentiviral cDNAs were produced in HEK293T cells using the trans-lentiviral ORF packaging kit with calcium phosphate (Dharmacon) following the manufacturer’s instructions. Viral particles were collected and concentrated. T-47D cells were infected, selected in presence of 1.5μg/mL of puromycin, and sorted by flow cytometry on the basis of the Tomato expression. Effective expressions of the cloned cDNAs were validated by Western blot (anti-Tomato: 1:1000, no. AB2722750, Sicgen). All Western blots were normalized by probing with an anti-actin (1:5000, no. AB10979409, Thermo Fisher Scientific).

### EVs isolation and characterization

Briefly, the different cell lines used to produce EVs were cultivated in absence of normocin and puromycin to reach 70% confluence, at which point their normal culture medium was changed to medium containing 10% FBS that had previously been depleted from calf serum EVs. To obtain appropriate cell medium without calf serum EVs, the appropriate cell medium was prepared with 20% FBS and centrifuged at 167,000 *g* for 18 h at 4 °C. After centrifugation the supernatant was collected, filtered through a 0.22μm membrane and kept at 4 °C until use. Cells were cultured in EV-free medium for 48 h, after which cell culture supernatant was collected for EVs isolation and a total cell lysate was obtained to compare total cell proteins composition to that of the EVs pellet (for EVs characterization purposes). EVs were isolated by differential centrifugation/ultracentrifugation techniques. First, the medium was centrifuged at 300 g for 10 minutes at 4 °C to remove cells. To remove dead cells, the supernatant was collected to be centrifuged at 2000 g for 20 minutes at 4 °C. In order to eliminate cell debris, the supernatant was collected to be centrifuged at 10,000 g for 30 minutes at 4 °C. The supernatant was collected to be ultracentrifuged in Ultra-Clear tubes using a 45Ti rotor (Beckman Coulter) at 120,000 g for 90 minutes at 4 °C in an Optima XPN-80 ultracentrifuge (Beckman Coulter). The supernatant was discarded and the remaining pellet, containing EVs and contaminating proteins, was washed twice in a large amount of cold PBS, and centrifuged again for 90 minutes at 120,000 g at 4 °C. This last ultracentrifugation was done a second time using fresh PBS to thoroughly eliminate the contaminating proteins. The pellet, containing the EVs fraction, was then resuspended in a minimal amount of cold PBS. The final EVs solution was aliquoted in siliconized polypropylene microcentrifuge tubes (Sigma, #T3281-500EA) and aliquots were kept at -80 °C until use. EVs were characterized by NTA (Nanoparticle Tracking Analysis) using the Nanosight (Malvern Instruments, Software version NTA 3.1 Build 3.1.46). PBS used for sample dilution was filtered and checked for vesicles absence prior to use. Calibration was performed using silica microspheres of 0.10μm sold at a known concentration of 4,897 × 10^13^ pp/mL (particles/mL) (Biovalley, #24041). Each sample was analyzed twice (using two different dilutions and aliquots) and 5 videos of 60 seconds were made per analysis. Final sample concentration was calculated as a mean of the different concentrations measured per analysis and as a proportion of the difference of concentration of the silica microspheres given by the Nanosight and the known concentration informed by the manufacturer. Total protein lysates from EVs were compared to that of their cells of origin by Western blot. Samples were prepared in non-reducing sample buffer with 2% SDS to EVs samples. 20 μg of protein were loaded per lane and separated with a 12.5% SDS-acrylamide gel. The antibodies used were: rabbit anti-human calnexin (1:100, no. AB2243890, Santa Cruz); mouse anti-human CD63 (1:1000, no. AB396297, BD Biosciences) and mouse anti-human CD81 (1:1000, no. AB2275892, Santa Cruz Biotechnology).

### Invasion assay

Cells were seeded in 12-wells plates and starved the following day by removing the FBS. Twenty-four hours later, medium or EVs was added to the wells as described in the figures. Twenty-four after adding the EVs, cells were trypsinized, resuspended in cell medium containing 1% BSA, centrifuged and washed once in free medium. Cells were then resuspended in medium containing 0.1% BSA and seeded in transwell chambers with 8μm pores (BD Falcon, #353097) in a 24-well companion plate previously coated with Matrigel (Corning, #356234 0.5 μg/insert). Conditioned medium isolated from NIH3T3 cells was added to the wells under the chambers and used as attractant for cell invasion. Cells were allowed to invade for different periods of time: 6 h for MDA-MB-231, SUM159PT, BXPC3, U-87MG, WM.266.4 and 24 h for T-47D and MCF7 cell lines Then, non-invading cells were removed from the top of the transwell with a cotton swab, whereas invading cells were fixed in 100% ethanol for 10 minutes and stained with hematoxylin (#HHS16-500mL, Sigma Aldrich) for 25 minutes. Within each experiment, each condition was tested in technical triplicates and the number of cells in the transwell whole field was counted to evaluate the cells’ ability to invade the Matrigel. Thus, the invasion index was calculated as the proportion of the number of invading cells in treated wells compared to the number of invading cells in the control wells (-) arbitrarily set to 1.

### Immunofluorescence

At the end of mice *in vivo* experiments (around 60 days after cell implantation in the fat pad), tumors dissection and frozen tumor tissues sections were performed. Briefly, slides were fixed in 4% paraformaldehyde, saturated for non-specific binding by incubation in blocking buffer (PBS, 1% BSA, 10% Donkey serum, 0.1% TX-100) for 1 h at room temperature. Then, the slides were incubated with specific antibodies to mouse macrophages anti-F4/80 antibody (1:100, no. AB1140040, Abcam) in blocking buffer overnight at 4 °C. The following day, the slides were washed and incubated with Alexa Fluor 594 Donkey anti-Rat IgG (H + L) Secondary Antibody (diluted 1:200, Abcam, #ab6640) in blocking buffer for 1 hour at room temperature. The slides were then washed, and non-specific binding was blocked in blocking buffer (PBS, 1% BSA, 10% mouse serum, 0.1% TX-100) for 1 h. The slides were then incubated for 1 h at room temperature with Anti-Pan human cytokeratins Alexa Fluor 488 antibody. After washes, slides were mounted in Fluoromount G mounting media with diluted DAPI (1:1000; no. AB259536, Sigma-Aldrich).

### Proliferation, apoptosis and spheroids growth detection

For proliferation and apoptosis detection, in a 2D cell system culture, cells were seeded in 96-well plates in their corresponding medium completed with 3% FBS. The following day the medium was removed and replaced with medium containing a fluorescent marker of activated caspase 3/7 (Green INCUCYTE caspase-3/7, Essen Bioscience, #4440) to evaluate apoptosis and a fluorescent nuclear marker to evaluate proliferation (INCUCYTE Nuclight Rapid Red, Essen Bioscience, #4717). Cells were either treated with EVs or with empty medium as described in the figures. Red and green fluorescent images of the cells were acquired every 2 hours for 4 days on the INCUCYTE device (Essen Bioscience). To evaluate proliferation and apoptosis, Areas Under the Curves (AUC) were calculated with the GraphPad Prism software for each tested condition for 4 days.

For spheroids growth and apoptosis detection, in a 3D cell system culture (spheroids: 5 × 10^3^ MDA-MB-231 or 5 × 10^3^ SUM-159-PT and hetero-spheroids: 5 × 10^3^ MDA-MB-231 + 2.5 × 10^3^ RAW264.7 or 5 × 10^3^ SUM-159-PT + 2.5 × 10^3^ RAW264.7), cells were seeded in a 96-well ultra-low attachment plates (Corning, #7007) in 100 μL in their corresponding medium containing 3% FBS and 3% Matrigel, and let for 3 days in the incubator to allow the spheroids to form. Then medium containing the fluorescent marker of activated caspase 3/7 (Green INCUCYTE caspace-3/7, Essen Bioscience) was added for 6 h to the wells in order to obtain a homogeneous labeling of the cells. Six hours later, medium containing EVs or not was added to the wells as described in the figures. Green fluorescent images were acquired on the INCUCYTE device (Essen Bioscience) every 2 hours for 4 days. AUC of spheroids, hetero-spheroids growth and apoptosis were calculated with the GraphPad Prism software for each tested condition for a total time of 4 days.

### *In vivo* experiments

Six-week old Hsd: Athymic Nude-Foxn1nu female mice were purchased from Charles Rivers company. Mice were hosted during 1 week at IRSL animal facility before they were manipulated. Animals were handled according to the guidelines of institutional animal care committees using protocols approved by the Comité d’Ethique en Experimentation Animale Paris-Nord. Mice were maintained in a 12-h light–dark cycle animal facility under specific pathogen–free conditions with free access to water and food. When the mice reached 7 weeks old, 5.10^5^ MDA-MB-231 cells (D3H2LN-LUC) were injected into the lower right left Fat Pad (8 or 10 mice/group). Tumor growth was evaluated by the tumor volume measure (cm^3^) twice week with a caliper. In this model, metastases began to arise from day 35, mainly axillary; twice a week, bioluminescent images were acquired on an IVIS system to quantify metastases burden. To this end, mice were intra-peritoneally injected with luciferin (150 mg/kg) diluted in DPBS 15 minutes before imaging. Fifteen minutes after luciferin injection, isoflurane anesthetized mice were placed on the back in the acquisition chamber of the IVIS system at 37 °C to record the luminescence produced by the axillary metastases for 1 second. To be able to visualize axillary metastases, the primary tumor was shielded with a black tissue to block the tumor high luminescence that would prevent the lower luminescence detection of the metastatic cells. The luminescence produced was quantified by the mean of photon flux (photons per second) produced by luciferase-positive metastatic cells. The photon flux is proportional to the number of light emitting cells. This method allows the follow-up of the same mice for the 2 months duration of the experiments and is closer to the clinical situation where metastases arise from a primary tumor. EVs (5.10^9^ pp in 100 μL of PBS) or PBS were injected in the tumor once a week for 2 months, otherwise as indicated in the legend of the figures. Mice were sacrificed by cervical dislocation.

### Microscope image acquisition

Acquisition and processing of images: Fluorescence images were captured using a Nikon eclipse Ti microscope, magnification 20×, PLAN APO X20 N.A 0.75; room temperature; Fluoromount G mounting media; with the fluorochromes Alexa Fluor 594, FITC and DAPI; with a Hamamatsu digital camera C11440; with the NIS version 4.20 acquisition software.

### Statistical analysis

The error bars in the graphical data represent the means ± SEM. When relevant, statistical significance was determined by a paired or unpaired two-tailed Student’s *t* test using GraphPad Prism software.

## Supplementary information

Supplementary information.

## Data Availability

No datasets were generated or analyzed during the current study.
