# Supplementary material for: Extracellular vesicles produced by NFAT3-expressing cells hinder tumor growth and metastatic dissemination
Source: Sci Rep. 2020 Jun 2;10:8964. doi: 10.1038/s41598-020-65844-x (PMC7265394; doi:10.1038/s41598-020-65844-x)

Extracellular vesicles produced by NFAT3-expressing cells hinder tumor growth and metastatic dissemination

Lívia Cardoso Bueno de Camargo[1], Frédéric Guaddachi[1], David Bergerat[2], Nadia Ourari[1], Lucie Coillard[1], Veronique Parietti[3], Morgane Le Bras[1], Jacqueline Lehmann-Che[1,4], and Sébastien Jauliac[1,*].

[1] Université de Paris, Research Saint Louis Institute (IRSL), INSERM HIPI U976, F-75010 Paris, France
[2] Inovarion SAS, (Paris, ile de France, France)
[3] Université de Paris, Saint-Louis Hospital, Research Saint Louis Institute (IRSL), Département d'Expérimentation Animale (Paris, ile de France, France)
[4] Molecular Oncology Unit, AP-HP, Hôpital Saint Louis, F-75010 Paris, France
[*] sebastien.jauliac@inserm.fr

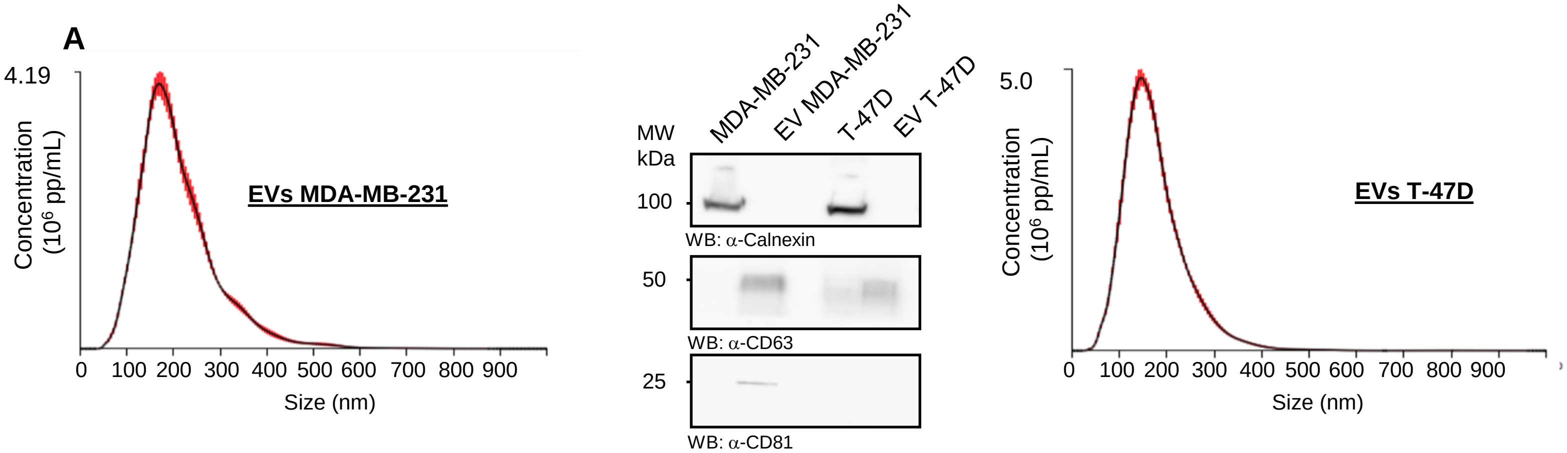

EVs producing cell line	Total number of cells	Total number of EVs produced (particles=pp)	Produced EVs number/cell
T-47D	50,4 x 10^8	12,8 x10^11	254
MDA-MB-231	4.5 x10^8	1 x 10^11	222

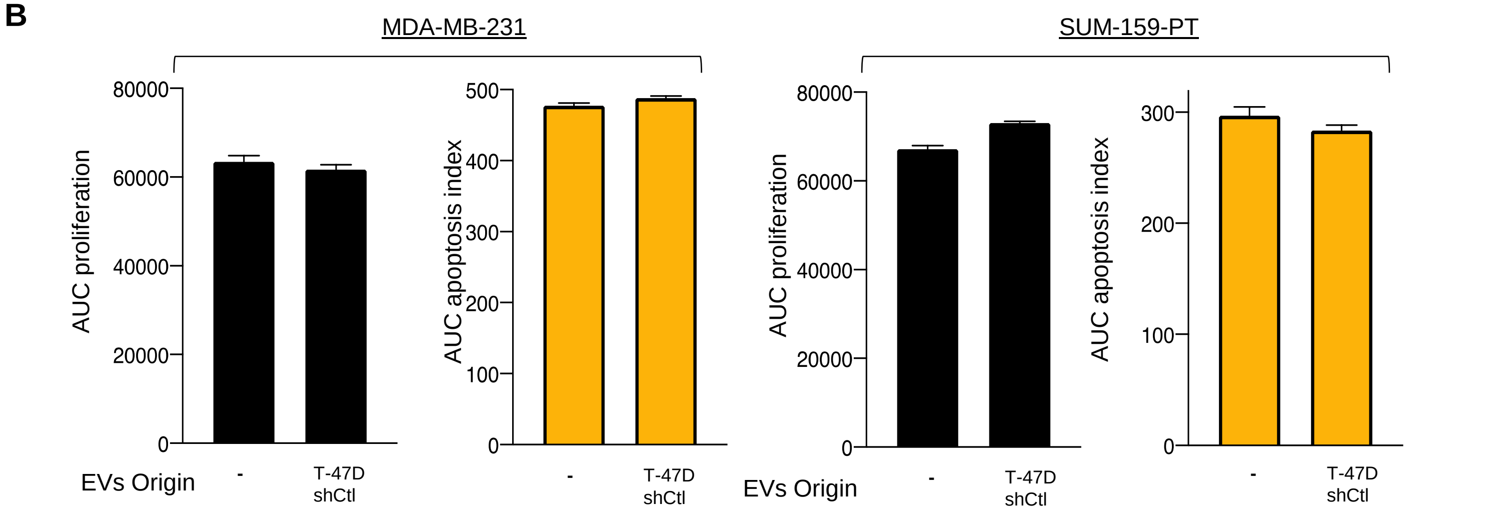

Figure S1. **Characterization of EVs produced by MDA-MB-231 and T-47D and effect of EV on cell proliferation and apoptosis. (A)** Representative examples of size distribution of EVs isolated from MDA-MB-231 (left panel) and T-47D (right panel), evaluated by NTA (Nanoparticle Tracking Analysis) with the NanoSight. Vesicles concentration (pp/mL) is shown in function of particle size (nm). Red error bars indicate +/-1 standard errors of the mean. Middle panel: Western blot for EVs characterization: total cell protein extracts were compared to total proteins level of the EVs fraction for both cell types using antibodies against Calnexin, CD63 and CD81. In the lower table are indicated representative EVs production with the total number of cells used/production, the total number of particles produced/production and the total number of produced EVS/cell. **(B)** Proliferation and apoptosis of INCUCYTE NucLight Rapid Red/fluorescent caspase 3/7 substrate co-labelled MDA-MB-231 and SUM-159PT were quantified on the INCUCYTE for 96 hours. Data from one representative experiment of three independent experiments is shown.

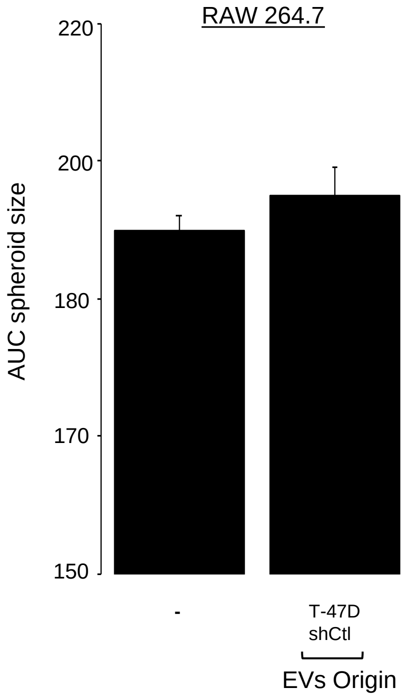

apoptosis undetectable

Figure S2. **EVs produced by T-47D cells do not modify the spheroid growth of the RAW 264.7** macrophages cell lines. RAW 264.7 macrophage cell line was plated in medium with 3% Matrigel in 96 ultra low attachment plates for 3 days to allow the formation of the spheroids. Medium was then added to each condition containing the apoptosis indicator (fluorescent caspase 3/7 substrate) supplemented with medium containing or not the EVs T-47D shCtl (3.108 pp/mL) as indicated in the figure. Size of the spheroids and appearance of green fluorescence (apoptosis) were recorded every 2h on an Incucyte apparatus for 4 days. Data are represented as the AUC of the spheroid size (left panel) and apoptosis was undetectable over 96h (n=3 technical replicates).

A

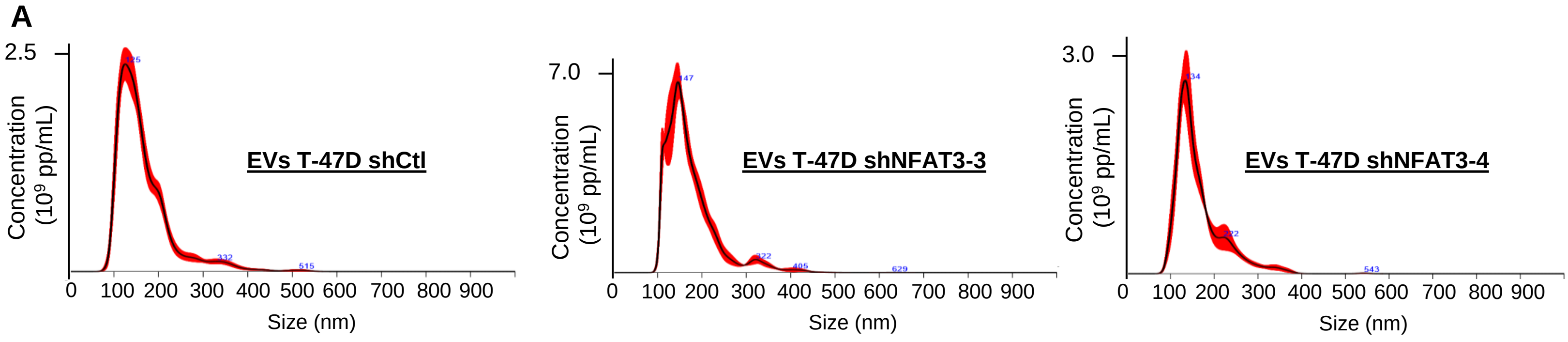

EVs producing cell line	Total number of cells	Total number of EVs produced (particles=pp)	Produced EVs number/cell
T-47D shCtrl	28.2 x 10^8	6.81 x10^11	241
T- 47D shNFAT3-3	29 x 10^8	5.56 x 10^11	192
T-47D shNFAT3-4	25.8 x 10^8	5.81 x 10^11	225

B

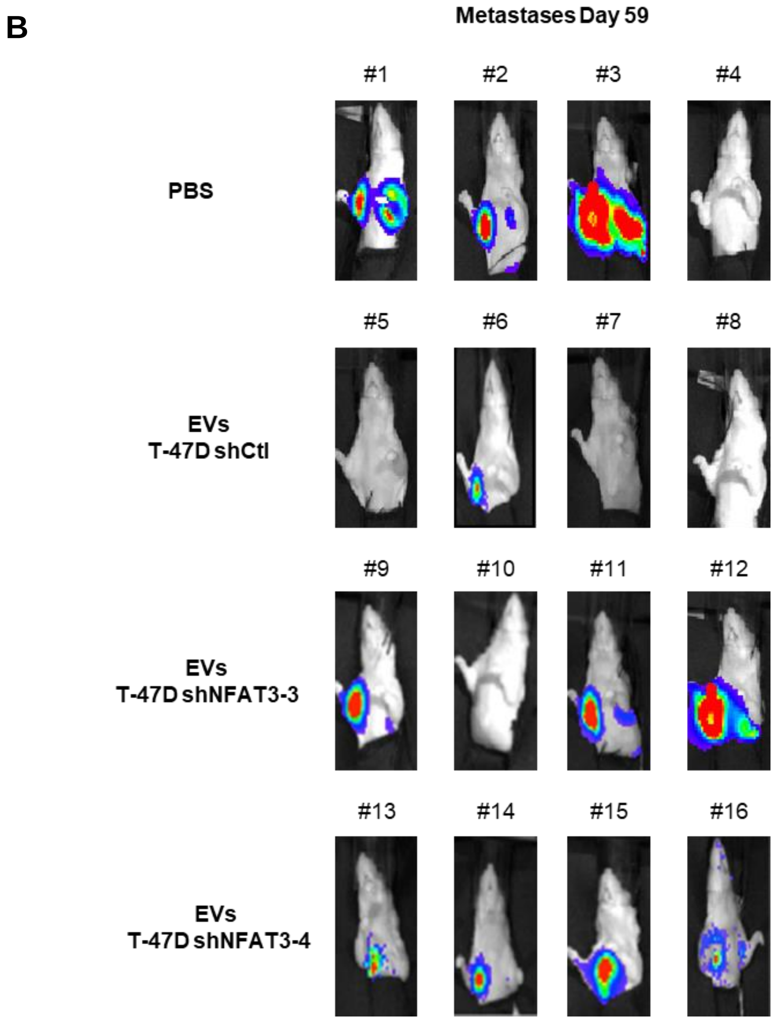

Figure S3**. Characterization of EVs produced by T-47D shCtl, shNFAT3-3, shNFAT3-4 and representative mice pictures of the anti-metastatic effect of EVs T-47D shCtl. (A)** Representative examples of size distribution of EVs isolated from T-47D shCtl (left panel), T-47D shNFAT3-3 (middle panel) and T-47D shNFAT3-4 (right panel) evaluated by NTA with the NanoSight. Vesicles concentration (pp/mL) is shown in function of particle size (nm). Red error bars indicate +/-1 standard errors of the mean. In the lower table are indicated representative EVs production with the total number of cells used/production, the total number of particles produced/production and the total number of produced EVs/cell. **(B)** Representative mice pictures acquired on the IVIS system of the anti-metastatic effect of EV T-47D shCtl at day 59 after D3HLN cells implantation in the fat pad.

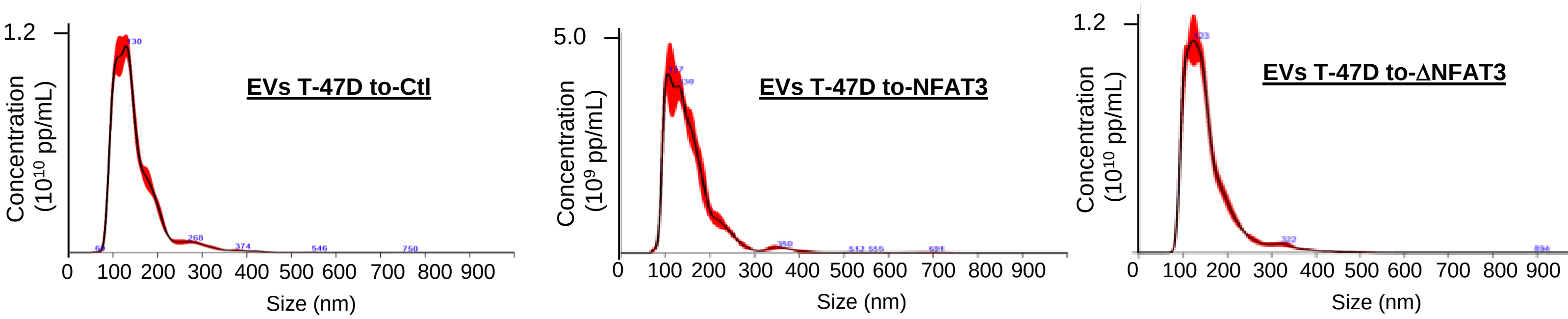

EVs producing cell line	Total number of cells	Total number of EVs produced (particles=pp)	Produced EVs number/cell
T-47D to-Ctl	34 x 10^8	11.12 x10^11	327
T-47D to-NFAT3	10.2 x10^8	2.39 x 10^11	284
T-47D to-ΔNFAT3	25.6 x 10^8	9.14 x 10^11	357

Figure S4**. Characterization of EVs produced by T-47D to-Ctl, to-NFAT3, to-ΔNFAT3.** Representative examples of size distribution of EVs isolated from T-47D to-Ctl (left panel), T-47D to-NFAT3 (middle panel) and T-47D to-ΔNFAT3 (right panel) evaluated by NTA with the NanoSight. Vesicles concentration (pp/mL) is shown in function of particle size (nm). Red error bars indicate +/-1 standard errors of the mean. In the lower table are indicated representative EVs production with the total number of cells used/production, the total number of particles produced/production and the total number of produced EVs/cell.

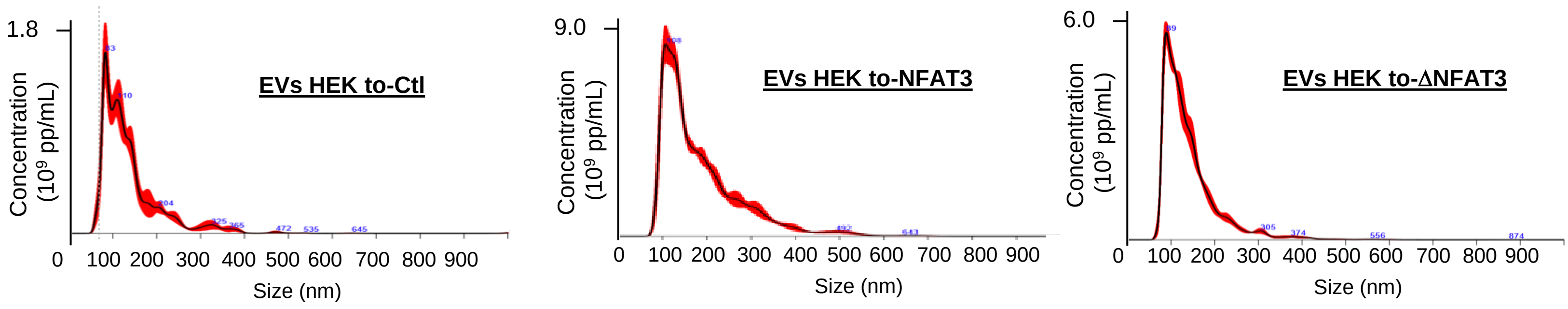

EVs producing cell line	Total number of cells	Total number of EVs produced (particles=pp)	Produced EVs number/cell
HEK to-Ctl	6.7 x 10^8	3.9 x10^11	582
HEK to-NFAT3	5.6 x 10^8	3.4 x 10^11	607
HEK to-DNFAT3	7.25 x 10^8	3.7 x 10^11	510

Figure S5**. Characterization of EVs produced by HEK to-Ctl, to-NFAT3, to-ΔNFAT3.** Representative examples of size distribution of EVs isolated from HEK to-Ctl (left panel), T-47D to-NFAT3 (middle panel) and T-47D to-ΔNFAT3 (right panel) evaluated by NTA with the NanoSight. Vesicle concentration (pp/mL) is shown in function of particle size (nm). Red error bars indicate +/-1 standard errors of the mean. In the lower table are indicated representative EVs production with the total number of cells used/production, the total number of particles produced/production and the total number of produced EVs/cell.

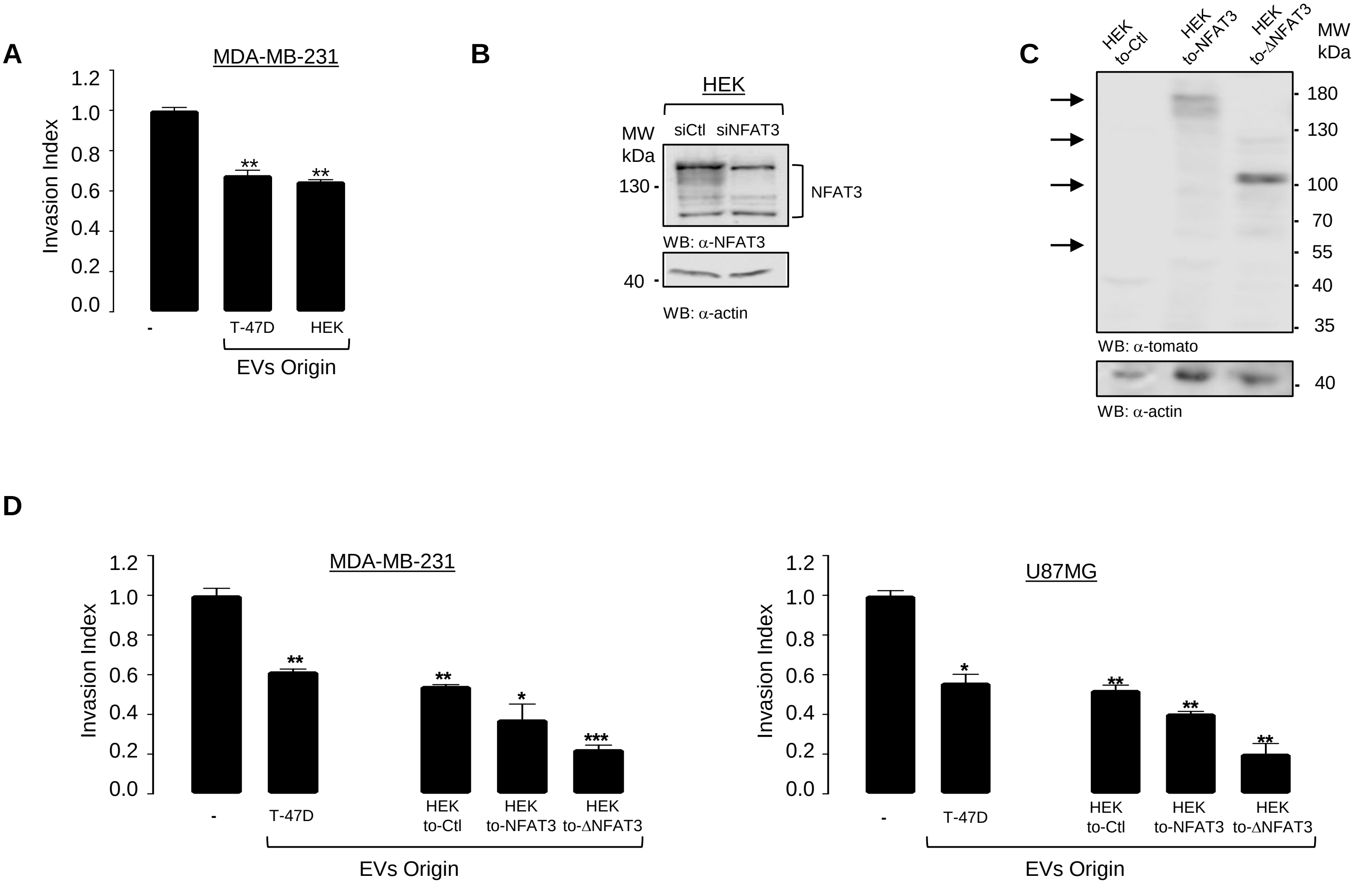

Figure S6. **Overexpression of NFAT3 in HEK293T cell is sufficient to increase the anti-invasive capacity in vitro of EVs produced by HEK293T cells. (A)** Highly invasive triple negative breast cancer cells MDA-MB-231 were serum starved for 24h and left untreated or were treated the following day with 3.108 pp/mL EVs isolated from by WT T-47D or from WT HEK cells and subjected to in vitro invasion assay for 6h. Data from one representative experiment of two independent experiments is shown, all data are shown as mean ± SEM (n=3 technical replicates; $**p<0.005$). **(B)** HEK293T (HEK) cells transiently transfected with control siRNA (siCtl) or siRNA directed against endogenous NFAT3 (siRNA NFAT3) with Dharmafect-1 for 48 h as indicated by the manufacturer; 48 h post-transfection whole cell lysates were revealed by an anti-NFAT3 (α-NFAT3) and normalization was done by revelation with an anti-actin (α-actin). **(C)** Stable clones of the HEK cell line were generated expressing lentiviral constructs encoding either the control vector with a Tomato tag (HEK to-Ctl) or the wild type NFAT3 (HEK toNFAT3) or the active N-terminal deletion mutant of NFAT3 fused to the Tomato tag (HEK to-ΔNFAT3). Whole cell lysates were revealed by an anti-tomato (α-tomato) and normalized by revelation with an anti-actin (α-actin) after stripping on the same blot. **(D)** Highly invasive MDA-MB-231 (right panel) and U87MG cells (left panel) were serum starved and pre-treated or not with 3 X 10^8 pp/mL EVs produced by WT T47D or by HEK to-Ctl, HEK toNFAT3 and HEK toΔNFAT3 and tested for their invasive capacity for 6h. Data from one representative experiment of three independent experiments is shown, all data are shown as mean ± SEM (n=3 technical replicates; $**p < 0.005$, $p<0.001$, compared to the untreated cells). For all data, the invasion index is calculated as a proportion of the number of invasive cells in treated wells compared to the number of invasive cells in the control well (-) arbitrarily set to 1.

Figure S7: **Full lenght unprocessed gels images**

Figure 2A

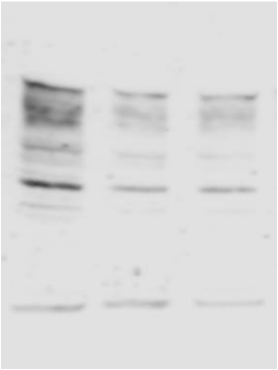

WB: α-NFAT3

WB: α-actin

Figure 4A

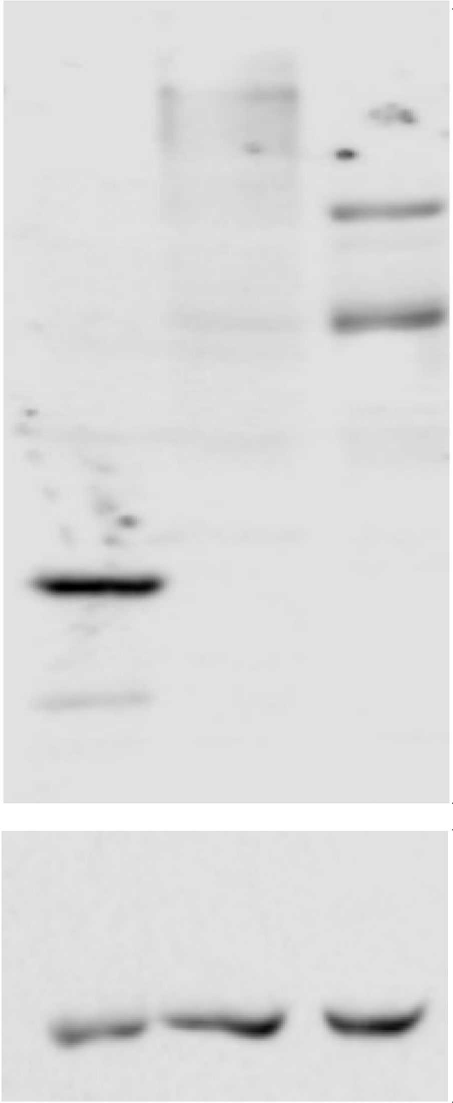

WB: α-tomato

WB: α-actin

Figure S6C

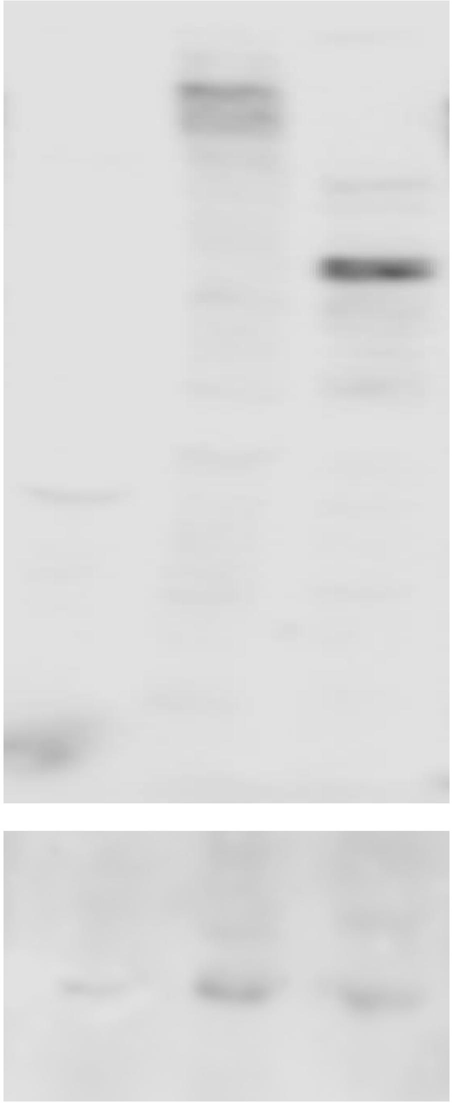

Figure S6B

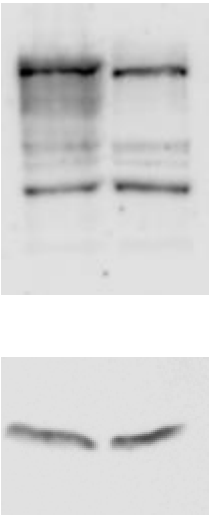

WB: α-NFAT3

WB: α-actin

Figure S1A

WB: α-Calnexin

WB: α-CD63

WB: α-CD81

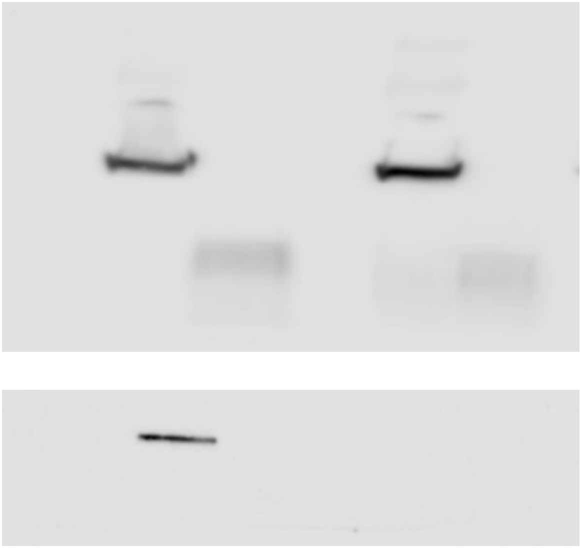

Supplement: Supplementary file 1 — Supplementary information. [file 41598_2020_65844_MOESM1_ESM.pdf]
